# The Effects of Alcohol, Nitro-Glycerine, and Amyl Nitrite in Shock

**Published:** 1904-05-28

**Authors:** 


### THE EFFECTS OF ALCOHOL, NITRO-GLYCERINE, AND AMYL NITRITE IN SHOCK.

The surgical world is very much indebted to Dr. G. Crile for his valuable experimental researches into the physiology of traumatic shock, the nature of which he has done perhaps more than anyone else to elucidate; and some further inquiries which he has made concerning the effects of certain drugs upon dogs in which a condition of shock had been induced will be received with considerable interest.[1] The drugs used, namely, whisky, nitro-glycerine and amyl nitrite, were all vaso-dilators, and they all had the effect of increasing shock.

In the case of whisky administered intravenously, the first effect usually noted was a decline in the blood pressure. In the majority of such instances a compensatory rise followed; in a number of instances no change in this respect was noted; in but few was there a rise in the blood pressure. The average height of the pulse-wave was increased. There was no evidence that the heart beat more forcibly. In animals reduced to varying degrees of surgical shock the usual effect of an average dose of whisky was the production of a further depression. In a few instances, in deep shock, the administration of a considerable dose was followed by almost immediate death, and the more profound the shock the more marked was the depressing effect of the whisky.

The immediate effect of nitro-glycerine and amyl nitrite upon the pulse was an increase in its volume and a decrease in frequency. The immediate effect upon the blood pressure was a fall, usually rapid, and followed by a gradual rise up to, in some cases, but rarely above, the level previous to the administration of the drug. If the doses were repeated, the succeeding effects resembled the preceding one, even when the repetitions were at short intervals. Very large repeated doses were tolerated. On the whole, nitro-glycerine and amyl nitrite increased shock. Although in the experiments with alcohol it is not stated what brand of whisky was used, Dr. Crile's experiments must be taken as evidence that the common practice of giving brandy, whisky and other alcoholic stimulants to counteract surgical shock is a misguided and possibly dangerous custom.

[1] Medical News, May 7.

